# Pten-Mediated Antioxidant Response Alleviates Hydrogen Peroxide-Induced Oxidative Damage in Tilapia Muscle

**DOI:** 10.3390/antiox15040499

**Published:** 2026-04-17

**Authors:** Jianxiang Chen, Pao Xu, Miaomiao Xue, Jiyan He, Huaishun Shen, Hongxia Li, Changyou Song

**Affiliations:** 1 Wuxi Fisheries College, Nanjing Agricultural University, Wuxi 214081, China; 2020113020@stu.njau.edu.cn (J.C.); xup@ffrc.cn (P.X.); 2021113016@stu.njau.edu.cn (M.X.); shenhuaishun@ffrc.cn (H.S.); 2 Key Laboratory of Freshwater Fisheries and Germplasm Resources Utilization, Ministry of Agriculture and Rural Affairs, Freshwater Fisheries Research Center, Chinese Academy of Fishery Sciences, Wuxi 214081, China; 2022813053@stu.njau.edu.cn

**Keywords:** oxidative stress, Pten, muscle, Nile tilapia

## Abstract

The mechanisms underlying hydrogen peroxide (HP)-induced oxidative stress damage in the muscle of Nile tilapia (*Oreochromis niloticus*) remain poorly understood. In this study, an oxidative stress model was established through 2 mM HP exposure for 4 weeks to elucidate the effects of oxidative stress on tilapia muscle and regulatory mechanisms. The results demonstrated that prolonged oxidative stress inhibited the antioxidant response in tilapia muscle and significantly reduced body weight. Concurrently, oxidative stress downregulated the gene expression of muscle proliferation and development, leading to a loss of muscle mass and the deterioration of muscle texture. Furthermore, oxidative stress altered muscle cell fate and exacerbated inflammatory responses. Further transcriptomic analysis revealed that Pten played a critical regulatory role in the muscle antioxidant response and growth. Mechanistically, activation of Pten ameliorated antioxidant capacity and promoted cell proliferation. In conclusion, HP-mediated oxidative stress significantly inhibited muscle proliferation and development, while targeted regulation of Pten effectively alleviated the suppression of muscle antioxidant capacity and cell proliferation. This study provided a theoretical basis for the prevention and control of oxidative stress injury in tilapia aquaculture.

## 1. Introduction

In modern farming practices, aquatic animals are continuously exposed to a range of biological, physical, and chemical stressors, which induce oxidative stress. It arises from an imbalance between the production of reactive oxygen species (ROS) and the capacity of the biological system to protect cells from ROS-induced damage [1]. Excessive accumulation of ROS leads to physiological disorders, oxidative modification of biological macromolecules, inflammation, immunosuppression, cell death including apoptosis, and various pathological conditions that underlie many diseases [2]. In response to the persistent challenge posed by ROS, aquatic animals have evolved a multi-layered and synergistic antioxidant defense system [3]. Superoxide dismutase (SOD) converts superoxide anion (O_2_^−^) into H_2_O_2_, while catalase (CAT) and glutathione peroxidase (GPx) are responsible for scavenging H_2_O_2_. Simultaneously, the non-enzymatic antioxidant system (e.g., GSH, vitamin C) provides broad direct protection. The enzymatic and non-enzymatic antioxidant systems support each other, working collectively to eliminate reactive oxygen species and restore redox homeostasis [3].

As the primary edible portion of fish, muscle tissue is rich in high-quality protein, polyunsaturated fatty acids (such as EPA and DHA), and essential trace elements [4]. Oxidative stress impairs both physiological metabolism and meat quality attributes in muscle tissue [5]. ROS induce mitochondrial dysfunction and activate the ubiquitin–proteasome system, leading to protein catabolism, which results in reduced muscle growth performance and even atrophy [6]. ROS and their secondary products oxidize myoglobin, causing muscle discoloration and browning [7]. Concurrently, oxidative stress damages the myofibrillar network structure and compromises cell membrane integrity, leading to decreased water-holding capacity, texture softening, and increased drip loss in muscle [8]. Furthermore, the oxidative rancidity of polyunsaturated fatty acids, coupled with protein degradation, generates undesirable flavor compounds and is accompanied by the loss of essential nutrients, ultimately affecting the sensory properties and nutritional value of aquatic products [9]. Therefore, studying oxidative stress in muscle tissue has direct economic and food safety implications. However, the mechanisms underlying oxidative damage in tilapia muscle remain insufficiently understood.

Pten (phosphatase and tensin homolog) is a critical tumor suppressor protein that primarily regulates cell proliferation, survival, and metabolism by antagonizing the PI3K/AKT signaling pathway [10]. Recent studies have revealed that the functional activity of Pten is closely associated with its redox status. Pten enhances the cellular capacity to scavenge reactive oxygen species (ROS) by stabilizing p53 and upregulating the expression of antioxidant enzymes [11]. However, when ROS accumulation exceeds a certain threshold, Pten activity is lost, thereby relieving its inhibition of the Pi3k/Akt pathway. This mechanism is recognized as a key node in cellular responses to oxidative signals [12,13]. Such redox regulation positions Pten as a potential molecular hub linking oxidative stress to compromised muscle homeostasis. In fish, research on Pten has primarily focused on zebrafish, where Pten has been shown to play important roles in embryonic development, tail fin fold regeneration, tumorigenesis, and inflammatory responses [14,15,16]. Studies in *Japanese flounder* have also demonstrated that microRNAs can target Pten to regulate apoptosis and autophagy, thereby alleviating viral infection [17]. Nevertheless, the regulatory role of Pten in fish muscle tissue under oxidative stress conditions remains poorly understood. Therefore, an in-depth characterization of Pten function will contribute to understanding the mechanisms underlying growth and quality regulation in teleost muscle under oxidative stress.

Hydrogen peroxide (HP) is one of the most extensively studied reactive oxygen species and is frequently employed to establish models of oxidative stress or damage [18]. Jia et al. [19] demonstrated that an intraperitoneal injection of 400 mM HP induced severe oxidative damage and inflammatory responses in the liver of tilapia within 72 h. Immersion exposure to 1 mM HP for 14 days triggered oxidative stress, endoplasmic reticulum (ER) stress, and apoptosis in various tissues of common carp (*Cyprinus carpio*) [20]. Prolonged HP exposure (1 mM, 14 days) has been shown to alter gill structure and disrupt ion transport in carp, thereby adversely affecting ion balance and osmoregulation [21]. Furthermore, He et al. [22] indicated that chronic HP exposure suppresses the antioxidant response and compromises the health of Chinese mitten crab (*Eriocheir sinensis*). Additionally, exposure to 100 μmol/L HP has been reported to induce oxidative stress and inhibit cellular activity in the intestinal epithelial cells of spotted sea bass (*Lateolabrax maculatus*) [23]. Therefore, this study employs HP treatment to induce oxidative stress, aiming to investigate its impact on muscle growth and development in tilapia and to elucidate the underlying molecular mechanisms. Our findings provide novel insights into the response mechanisms to oxidative stress in fish muscle.

## 2. Materials and Methods

### 2.1. Ethics Statement

This study was approved by the Animal Care and Use Committee of Nanjing Agricultural University (Nanjing, China) in August 2024. All animal procedures were performed according to the Guideline for the Care and Use of Laboratory Animals in China.

### 2.2. Experimental Animals

Healthy male Nile tilapia (*Oreochromis niloticus*) (*n* = 80, 42.34 ± 1.60 g) were obtained from the Freshwater Fisheries Research Center, Chinese Academy of Fishery Sciences. The fish were reared in a recirculating aquaculture system under controlled conditions: water temperature 28 ± 1 °C, dissolved oxygen ≥ 5 mg/L, and a 12 h light: 12 h dark photoperiod. They were fed daily at 3% of their body weight and acclimated for one week before the experiment. The fish were fed on commercial diet containing 28% crude protein, 5% crude lipid, 15% crude ash, 12% crude fiber, 0.6% P and 12.5% water (Tongwei Co., LTD., Chengdu, China). During the experimental period, fish were randomly exposed to three concentrations of hydrogen peroxide (Macklin, China): 0 (control, Con), 2, and 7 mM (*n* = 18 per group; three replicates per group). Fish were treated with HP at the respective concentrations for 1 h daily over four weeks. The experimental concentrations were selected based on our preliminary test results, which showed that the 1 h median lethal concentration (LC50) for fish (45–65 g) was approximately 10 mM. Throughout the experiment, fish were fed once daily with a commercial diet at 1% of their body weight. At the end of the experiment, nine fish were randomly sampled from each group and immediately anesthetized with MS-222 (100 mg·L^−1^). The blood and dorsal muscles were collected. Serum was separated from blood by centrifugation at 4000 rpm for 10 min at 4 °C. Muscle samples of appropriate size were fixed in 4% paraformaldehyde and 2.5% glutaraldehyde. The remaining muscle samples were immediately frozen in liquid nitrogen and stored at −80 °C for subsequent analyses.

### 2.3. Muscle Texture Determination

Muscle texture properties were analyzed using an MS-Pro Texture Analyzer (Food Technology Corporation, Sterling, VA, USA) through texture profile analysis (TPA) mode. The test protocol employed a 50 mm diameter aluminum probe with two consecutive compression cycles. The pre-test speed was 5 mm/s, the test speed 1 mm/s, and the post-test speed 5 mm/s. Texture parameters such as hardness, springiness, chewiness, and cohesiveness were calculated from the force–time curve.

### 2.4. Physiological and Biochemical Analyses

The serum and muscle samples were used to determine malondialdehyde (MDA, A003-1-2) using thiobarbituric acid (TBA) method, catalase (CAT, A007-1-1) using ammonium molybdate method, total superoxide dismutase (T-SOD, A001-3-2) using WST-1 method, total antioxidant capacity (T-AOC, A015-2-1) using ABTS method and glutathione (GSH, A006-2-1) using microplate method. Detailed procedures refer to the manufacturer’s instructions. All detection kits were purchased from Jiancheng Bioengineering Institute (Nanjing, China). The specific steps for each assay were as follows: For MDA, according to the tube type (blank, standard, assay, control), the corresponding reagents/samples were added in sequence (absolute ethanol to the blank tube, standard to the standard tube, sample to the assay/control tubes, and Reagent 1 to all tubes), mixed, then Working Solution I/II was added and mixed again. Holes were poked in the caps of centrifuge tubes, followed by a water bath at 95 °C for 80 min and cooling under running water. After centrifugation at 3500 rpm for 10 min, the supernatant was collected and its absorbance measured at 532 nm. For CAT, Reagent 1 and Reagent 2 were added first. For the assay tube, the sample was added in advance, and after exactly 60 s of reaction, Reagent 3 was immediately added to terminate the reaction. For the control tube, the sample was added at the end without strict timing. Reagent 4 was then added to both tubes and mixed. The absorbance of each tube was measured at 405 nm. For T-SOD, sample, distilled water, enzyme working solution/diluent, and substrate application solution were added, vortexed to mix, and incubated for 20 min. The absorbance of each well was measured at 450 nm using a microplate reader. For T-AOC, Trolox standard solution was diluted to 0.1, 0.2, 0.4, 0.8, and 1.0 mM, then Reagent 4 application solution and ABTS working solution were added. After reacting at room temperature for 6 min, the OD was measured at 405 nm to fit a standard curve. Blank, standard, and sample wells were set up, and distilled water/standard solution/test sample, Reagent 4 application solution, and ABTS working solution were added according to the table. After reacting at room temperature for 6 min, the OD at 405 nm was measured. The corrected OD value was substituted into the standard curve formula to obtain the corresponding concentration. For GSH, blank, standard, and sample wells received Reagent 1, 20 μmol/L standard solution, and sample supernatant, respectively, followed by Reagent 3 and Reagent 2. After vortexing and standing for 5 min, the absorbance was measured at 420 nm.

### 2.5. Histological Analysis

The muscles were fixed in 4% paraformaldehyde, embedded in paraffin, and sectioned at a thickness of 5 μm for hematoxylin–eosin (H&E) staining.

### 2.6. Ultrastructure

The muscles from each group (*n* = 3) were fixed in 2.5% glutaraldehyde and stored at 4 °C. The specimens were embedded in epoxy resin (Epoxy Embedding Medium Kit, Sigma, Germany), then sectioned into 70 nm ultrathin slices using a Leica Ultra-cut UCT25 ultramicrotome (Wetzlar, Germany). After staining with uranyl acetate and lead citrate, the sections were observed under a Hitachi HT7700 transmission electron microscope (Hitachi, Tokyo, Japan).

### 2.7. RNA Sequencing

Total RNA was extracted from tilapia muscle tissues (*n* = 3, three samples per group) using TRIzol Reagent according to the manufacturer’s instructions. Then, RNA quality was determined by 5300 Bioanalyser (Agilent, Santa Clara, CA, USA) and quantified using the ND-2000 (NanoDrop Technologies, Wilmington, DE, USA). Only high-quality RNA sample (OD260/280 = 1.8–2.2, OD260/230 > 2.0, RQN ≥ 6.5, 28S:18S ≥ 1.0, >1 ug) was used to construct sequencing library at Shanghai Majorbio Bio-pharm Technology Co., Ltd (Shanghai, China). Sequencing was performed on the Illumina NovaSeq platform (San Diego, CA, USA) (https://support.illumina.com/sequencing/sequencing_instruments/novaseq-x-novaseq-x-plus/documentation.html, accessed on 18 March 2026) to generate raw reads. After quality control, the reads were aligned to the tilapia genome. The clean data for each sample reached at least 5.7 Gb, with the percentage of Q30 bases exceeding 96.14%. Differentially expressed genes (DEGs) were screened using DESeq2 software with the criteria of |log2 fold change| > 1 and *p* < 0.05. Enrichment analysis was conducted using the Gene Ontology (GO) and Kyoto Encyclopedia of Genes and Genomes (KEGG) databases.

### 2.8. ZF4 Cell Culture

Given the absence of a commercially available, stable Nile tilapia fibroblast cell line, ZF4 was selected due to its well-defined characteristics, high transfection efficiency, and its status as a widely accepted teleost model for cellular and molecular assays [24]. ZF4 was purchased from the Institute of Hydrobiology, Chinese Academy of Sciences. Cells were cultured in DMEM/F12 medium (11320033, Gibco, Waltham, MA, USA) supplemented with 10% fetal bovine serum (A5256701, Gibco) and 1% penicillin–streptomycin, and maintained in a constant-temperature incubator at 28 °C with 5% CO_2_. The Vo-ohpic (S8174, Selleck, Houston, TX, USA) and Oroxin B (S9190, Selleck, Houston, TX, USA) were dissolved in DMSO. The dissolved reagent was added to the culture medium at concentrations of 0.5, 1, 2, 4, and 8 μM.

### 2.9. Cell Viability Assay

ZF4 cells were seeded in 96-well plates and treated with different doses of HP. Cell viability was detected using the Cell Counting Kit-8 (CCK-8, CA1211, Solarbio, Beijing, China).

### 2.10. RNA Extraction and Real-Time PCR

Total RNA was extracted from muscles or ZF4 cells using RNAiso Plus reagent (Takara Co. Ltd., Dalian, China) followed by a series of chloroform-involved procedures. Complementary DNA (cDNA) was synthesized from 1 μg of extracted RNA using the PrimeScript™ RT reagent kit (TaKaRa). The expression levels of target genes were detected by quantitative real-time PCR (qRT-PCR) using TB Green™ Premix Ex Taq™ II Kit (TaKaRa). β-actin was used as the reference gene for normalizing CT values. The relative expression levels of target genes were analyzed using the 2^−ΔΔCT^ method. The sequences of primers used for qPCR analysis are listed in Supplementary Table S1.

### 2.11. Reactive Oxygen Species (ROS) Detection

ZF4 cells were stained with dihydroethidium (DHE, S0063, Beyotime, Shanghai, China). The level of ROS was reflected by the intensity of red fluorescence, which was observed under a fluorescence microscope.

### 2.12. EdU Staining Assay

ZF4 cells were seeded in 12-well plates. 5-Ethynyl-2′-deoxyuridine (EdU, C0071S, Beyotime, China) was added to each well, followed by incubation for 2 h. The cells were fixed with 4% paraformaldehyde. The cells were then incubated with PBS containing 0.3% Triton X-100 for 10 min. Click reaction solution was added according to the manufacturer’s instructions. After discarding the washing solution, the cells were stained with Hoechst 33342 (Beyotime) for 10 min at room temperature in the dark.

### 2.13. Statistical Analysis

One-way analysis of variance (ANOVA) was used for comparisons among multiple groups, and Student’s *t*-test was applied for comparisons between two groups. Data are presented as mean ± standard deviation (with at least *n* = 3). Statistical analyses were performed using Prism software (GraphPad Prism 5.0, San Diego, CA, USA), and a *p*-value < 0.05 was considered statistically significant. Additionally, Spearman correlation analysis was performed to determine the correlation between the antioxidants and physiological indicators.

## 3. Results

### 3.1. Long-Term Low-Concentration Hydrogen Peroxide (HP) Treatment Induces Oxidative Stress in Nile Tilapia Muscle

**Figure 1:**
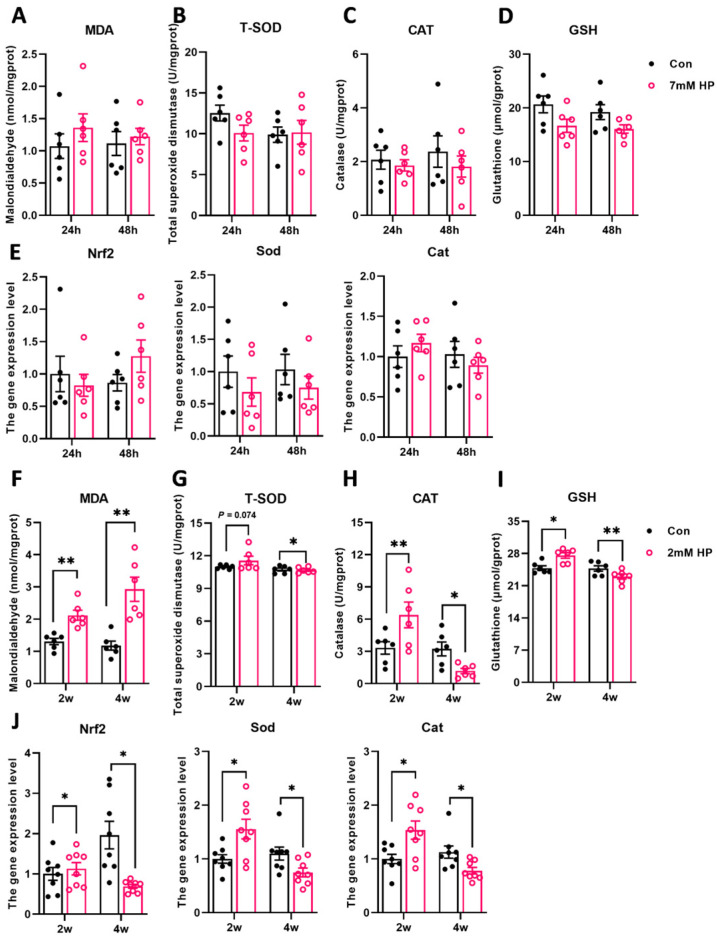
Long-term low-concentration hydrogen peroxide treatment induces oxidative stress in Nile tilapia muscle. Changes in antioxidant indicators under acute hydrogen peroxide (HP) treatment. (**A**) Malondialdehyde (MDA) content, (**B**) total superoxide dismutase (T-SOD) content, (**C**) catalase (CAT) content, (**D**) glutathione (GSH) content (*n* = 6), (**E**) gene expressions of antioxidant under acute HP treatment (*n* = 8). Changes in antioxidant indicators under chronic HP treatment. (**F**) Malondialdehyde (MDA) content, (**G**) total superoxide dismutase (T-SOD) content, (**H**) catalase (CAT) content, (**I**) glutathione (GSH) content (*n* = 6), (**J**) gene expressions of antioxidant under chronic HP treatment (*n* = 8). Results are expressed as mean ± SEM. * stands for *p* < 0.05 and ** stands for *p* < 0.01.

To investigate the effects of oxidative stress on tilapia muscle, we compared the antioxidant responses under short-term high-concentration (7 mM, 48 h) and long-term low-concentration (2 mM, 4 w) hydrogen peroxide treatments. In contrast to long-term stress, acute stress treatment did not induce oxidative stress in the muscle (Figure 1A–E). Conversely, 2 mM HP exposure for 4 weeks induced muscle oxidative stress. Specifically, MDA content in the 2 mM HP treatment group was significantly higher than in the control group at both 2 and 4 weeks, with a greater increase observed at 4 weeks (Figure 1F). A 2-week exposure stimulated the muscle antioxidant response, manifested as elevated levels of T-SOD, CAT, and GSH. However, long-term stress compromised the muscle’s antioxidant capacity and suppressed its antioxidant levels (Figure 1G,H). Similarly, at the molecular level, the gene expression of antioxidants (Nrf2, Sod, Cat) was also downregulated under prolonged stress (Figure 1J). These results indicated that HP exposure led to oxidative stress in tilapia muscle, resulting in the depletion of antioxidant substances and the exacerbation of lipid peroxidation.

### 3.2. Oxidative Stress Inhibited the Muscle Proliferation and Development of Nile Tilapia

**Figure 2:**
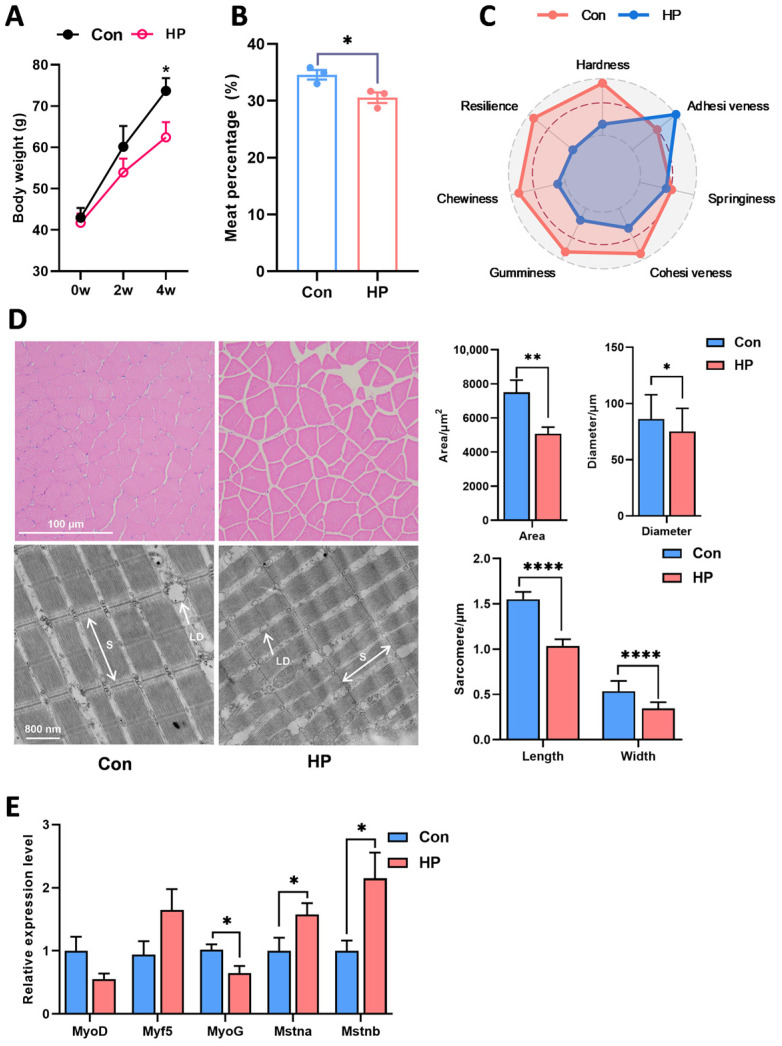
Oxidative stress inhibited the muscle proliferation and development of Nile tilapia. (**A**) Body weight under HP exposure. (**B**) Muscle content, (**C**) muscle texture, (**D**) changes in muscle tissue structure under HP treatment, S represents sarcomere length, and LD denotes lipid droplets (**E**) gene expression of muscle growth and development (*n* = 6). Myogenic differentiation (MyoD); myogenic factor 5 (Myf5); myogenin (MyoG); myostatin a (Mstna); myostatin b (Mstnb). Results are expressed as mean ± SEM. * stands for *p* < 0.05, ** stands for *p* < 0.01, **** stands for *p* < 0.0001.

Based on preliminary findings, 2 mM HP exposure for 4 weeks was selected as the experimental condition to induce muscle oxidative stress. As shown in Figure 2A, chronic HP exposure significantly inhibited the body weight gain of Nile tilapia. Consistent with this, the muscle mass of the HP-treated group was significantly lower than that of the control group (Figure 2B). Furthermore, chronic HP exposure significantly altered the muscle quality of Nile tilapia. Specifically, the hardness, chewiness, and elasticity of the muscle in the HP-treated group were significantly reduced, while adhesiveness slightly increased, indicating impaired muscle texture (Figure 2C). Histological analysis revealed that HP exposure led to a decrease in muscle fiber diameter and cross-sectional area. The inter-fiber spacing increased, and the arrangement became looser. Similarly, transmission electron microscopy results confirmed that the sarcomere length and myofibril width were reduced in the HP group (Figure 2D). Real-time quantitative PCR results also showed that the HP treatment downregulated gene expression associated with muscle growth and development, including the inhibition of MyoG and the activation of Mstn (Figure 2E). In summary, HP-induced oxidative stress in the muscle caused structural damage and inhibited muscle growth and development.

### 3.3. Oxidative Stress Induced Autophagy, Apoptosis, and Inflammation in the Nile Tilapia Muscle

**Figure 3:**
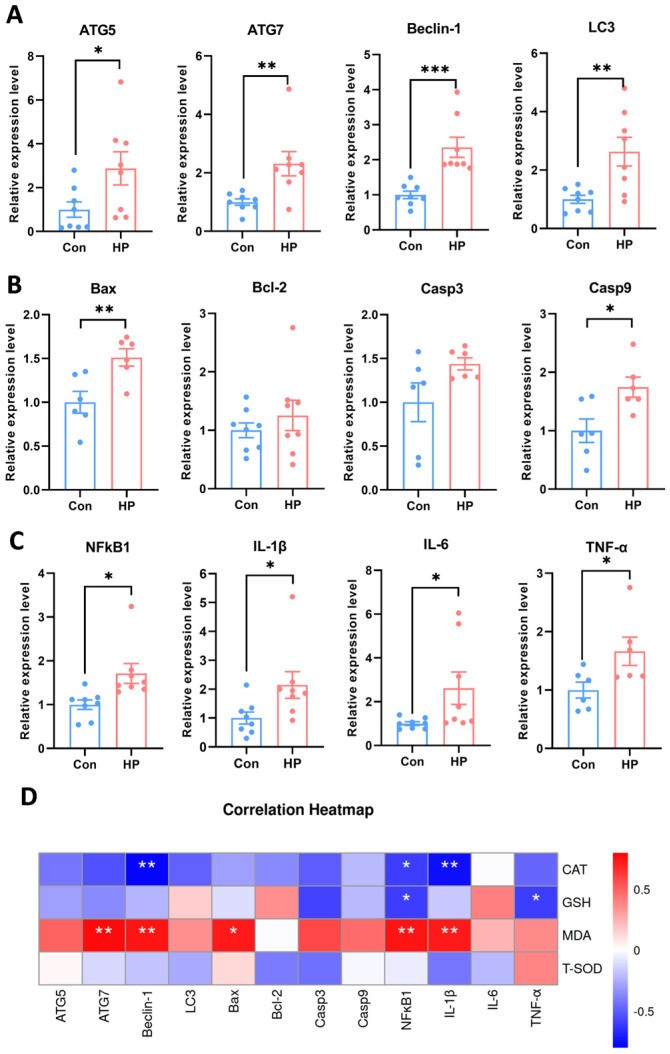
Oxidative stress induced autophagy, apoptosis and inflammation in the Nile tilapia muscle. (**A**) The gene expression of autophagy under HP exposure, *n* = 8. Autophagy related 5, ATG5; autophagy related 7, ATG7; Beclin-1; microtubule-associated proteins 1A/1B light chain 3, LC3. (**B**) The gene expression of apoptosis under HP exposure, *n* = 8. BCL2 associated X, Bax; B-cell lymphoma-2, Bcl-2; caspase-3, Casp3; caspase-9, Casp9. (**C**) The gene expression of inflammation under HP exposure, *n* = 8. Nuclear factor kappa-B1, NFκB1; interleukin-1β, IL-1β; interleukin-6, IL-6; tumor necrosis factor-alpha, TNF-α. (**D**) Correlation analysis between gene expression and antioxidant enzyme activity. Results are expressed as mean ± SEM. * stands for *p* < 0.05, ** stands for *p* < 0.01, *** stands for *p* < 0.001.

Chronic HP exposure not only affected muscle structure but also significantly altered the physiological state of Nile tilapia muscle. The expression of autophagy-related genes ATG5, ATG7, Beclin-1, and LC3 was significantly upregulated in the HP-treated group (Figure 3A), indicating that oxidative stress activated the autophagic response in muscle cells. Moreover, chronic exposure promoted the gene expression of apoptosis (Figure 3B). Additionally, oxidative stress triggered an inflammatory response in Nile tilapia muscle (Figure 3C). Further correlation analysis revealed a significant relationship between antioxidants and autophagy, apoptosis, and inflammation (Figure 3D). Specifically, MDA content showed positive correlations with the autophagy gene ATG7, Beclin-1, the pro-apoptotic gene Bax, and inflammatory factors such as IL-1β and TNF-α, while CAT and GSH activities showed negative correlations. This suggests that the suppression of the antioxidant system may be a key factor in triggering the development of these pathological processes.

### 3.4. Transcriptomic Analysis Reveals Molecular Mechanisms Related to Oxidative Stress

**Figure 4:**
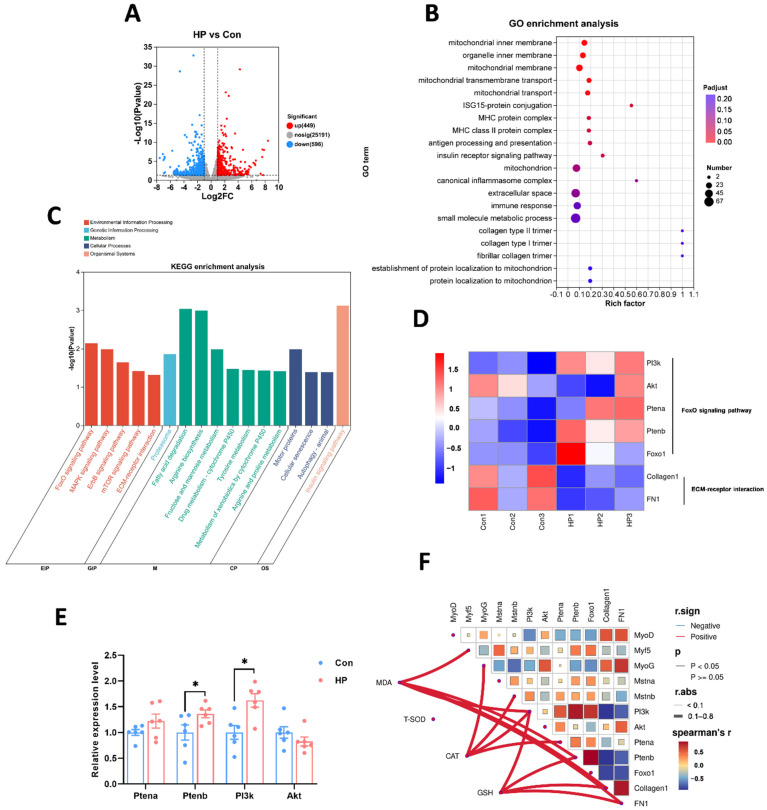
Transcriptome analysis under HP exposure in Nile tilapia muscle. (**A**) Volcano plot of DEGs in the muscle. (**B**) Bubble diagram demonstrates GO term enrichment analysis of the DEGs between HP and Con groups. (**C**) KEGG enrichment of DEGs in HP vs Con. (**D**) Hierarchical clustering of DEGs. (**E**) The gene expression of key DEGs under HP exposure, * stands for *p* < 0.05. (**F**) Correlation analysis between key genes and antioxidant enzyme. Red indicates positive correlations; solid line indicates *p* < 0.05.

To further uncover the mechanism of oxidative stress in tilapia muscle, we performed high-throughput sequencing to identify dynamically regulated mRNAs. Transcriptomic analysis revealed a large number of differentially expressed genes (DEGs) between the HP-treated and control groups, including 449 upregulated and 596 downregulated genes (Figure 4A). These DEGs were primarily enriched in biological processes such as mitochondrial transport, antigen processing and presentation, immune response, and inflammasome formation, as well as cellular components including the mitochondrial membrane and MHC protein complex (Figure 4B). KEGG enrichment analysis was also utilized for DEGs. The DEGs were significantly enriched in pathways such as the FoxO signaling pathway, ECM–receptor interaction, fatty acid and amino acid metabolism, cellular senescence, and autophagy (Figure 4C). Transcriptomic and RT-PCR validation results identified PI3K, Akt, Ptena, Ptenb, and genes in the ECM–receptor interaction pathway such as Colagen1 and FN1 were key DEGs under oxidative stress (Figure 4D,E). Correlation analysis showed a clear relationship between Pten and antioxidant enzyme activities (MDA, CAT, GSH), suggesting that Pten may affect oxidative stress injury by regulating the function of the antioxidant system (Figure 4F). Furthermore, Colagen1 and FN1 showed a significant negative correlation with Ptenb and a positive correlation with muscle proliferation and development, implying that the activation of the ECM–receptor interaction pathway was involved in repairing oxidative stress-induced muscle damage.

### 3.5. Inhibition of Oxidative Stress Ameliorated Hydrogen Peroxide-Induced Reduction in Cell Proliferation Capacity

**Figure 5:**
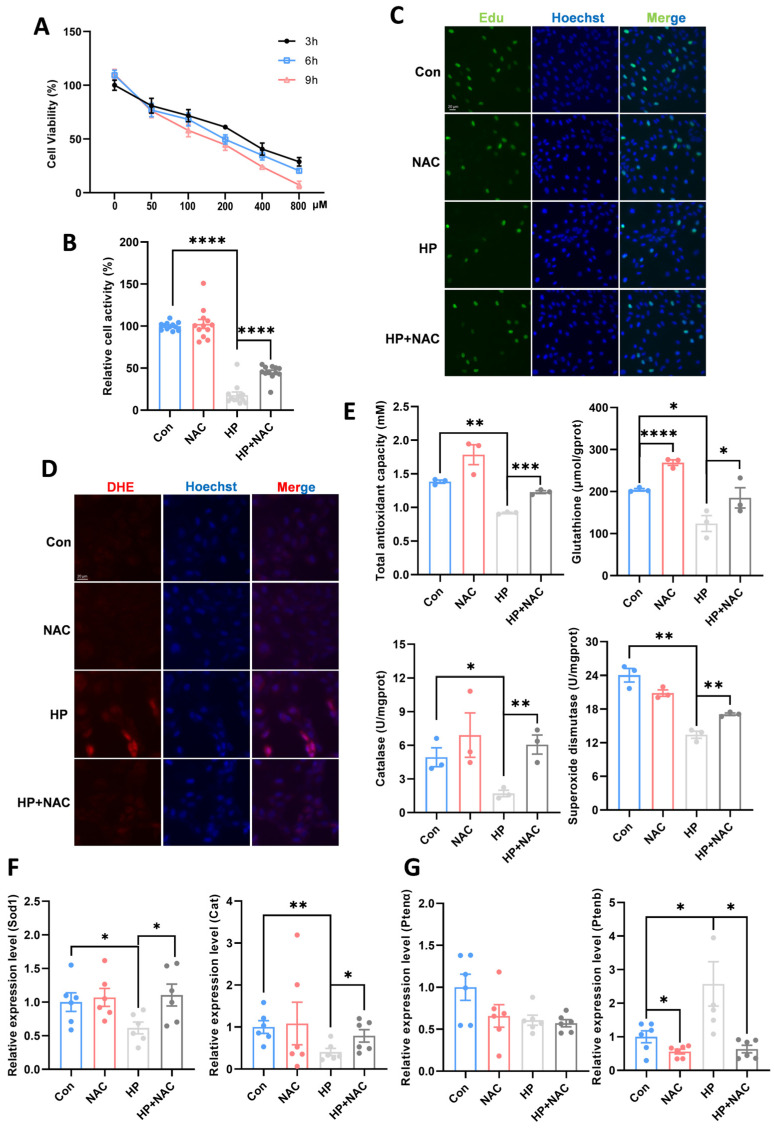
Inhibition of oxidative stress ameliorated hydrogen peroxide-induced reduction in cell proliferation capacity. (**A**) The cell viability during 9 h under different concentrations HP exposure (*n* = 12). (**B**) The cell viability during 9 h under NAC treatment (*n* = 12). (**C**) Edu staining (*n* = 3). (**D**) Reactive oxygen species (ROS) assay (*n* = 3). (**E**) Changes in antioxidant indicators under HP treatment. (**F**) The gene expression of antioxidant under HP exposure. (**G**) The gene expression of Ptena and Ptenb under HP exposure. * stands for *p* < 0.05, ** stands for *p* < 0.01, *** stands for *p* < 0.001, **** stands for *p* < 0.0001.

To determine the impact of oxidative stress, we conducted in vitro studies using the zebrafish ZF4 cell line. Cell viability assays showed that high-concentration, long-duration oxidative stress exposure caused more severe inhibition of cell viability (Figure 5A). Based on these results, we selected 400 µM HP treatment for 3 h to induce cellular oxidative stress. NAC, as an antioxidant, significantly alleviated the HP-induced decrease in cell viability (Figure 5B). Edu staining results showed that the cell proliferation was significantly suppressed in the HP-treated group, whereas it was significantly increased in the HP+NAC group (Figure 5C), indicating that NAC can improve cell proliferation capacity by inhibiting oxidative stress. ROS detection showed that HP treatment significantly elevated intracellular ROS levels in ZF4 cells, and NAC treatment significantly reduced ROS (Figure 5D). Correspondingly, NAC also increased antioxidant enzyme activities and related gene expression (Figure 5E,F), demonstrating that enhancing antioxidant capacity to scavenge ROS can mitigate the effects of oxidative stress. Furthermore, it was notably observed that antioxidant treatment also decreased the expression of Ptenb (Figure 5G), once again hinting at the key role of Pten in oxidative damage repair.

### 3.6. Activation of Pten Ameliorated Hydrogen Peroxide-Induced Reduction in Cell Proliferation Capacity

**Figure 6:**
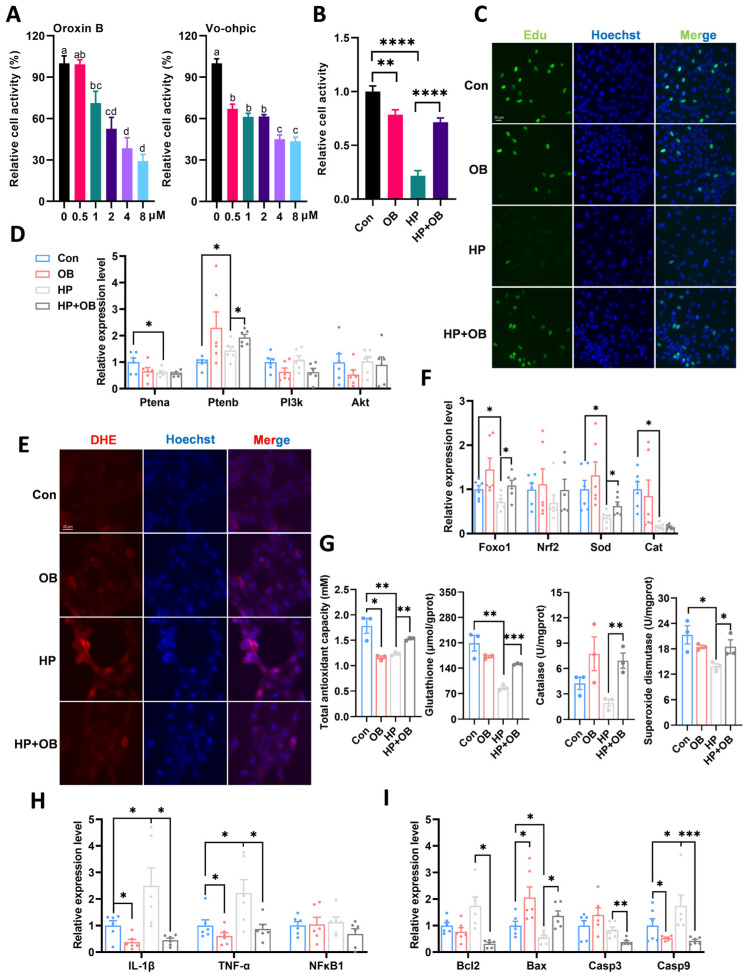
Activation of Pten ameliorated hydrogen peroxide-induced reduction in cell proliferation capacity. (**A**) The cell viability during 9 h under different concentrations Pten effector exposure (*n* = 12), different letters indicate significant differences, *p* < 0.05. (**B**) The cell viability during 9 h under OB and HP treatment (*n* = 12). (**C**) Edu staining (*n* = 3). (**D**) The gene expression of Pten (*n* = 6). (**E**) Reactive oxygen species (ROS) assay (*n* = 3). (**F**) The gene expression of antioxidation (*n* = 6). (**G**) Changes in antioxidant indicators under OB treatment. (**H**) The gene expression of inflammation under OB exposure. (**I**) The gene expression of apoptosis under OB treatment. * stands for *p* < 0.05, ** stands for *p* < 0.01, *** stands for *p* < 0.001, **** stands for *p* < 0.0001.

To further investigate the association between Pten and oxidative stress, regulators of Pten were applied. Vo-ohpic (VO, inhibitor) treatment significantly reduced cell viability, and similar results were observed with Oroxin B (OB, activator) treatment, but no significant change was noted at 0.5 µM OB (Figure 6A). Furthermore, treatment with this concentration of OB significantly improved cell viability after HP exposure (Figure 6B). EdU staining results indicated that OB treatment significantly restored the HP-induced inhibition of cell proliferation (Figure 6C). Gene expression analysis showed that OB treatment activated Pten gene expression (Figure 6D). Concurrently, activation of Pten significantly reduced HP-induced ROS production (Figure 6E). It was also confirmed that OB treatment upregulated the gene expression of antioxidation (Figure 6E) and improved the activities of antioxidant enzymes (Figure 6G). Additionally, activation of Pten markedly downregulated the expression of apoptosis- and inflammatory factor-related genes (Figure 6H,I). This indicated that activating Pten suppressed oxidative stress to ameliorate cell proliferation and cellular damage.

## 4. Discussion

In contemporary intensive aquaculture systems, fish are continuously exposed to multiple environmental and physiological stressors, rendering oxidative stress a significant impediment to their health and product quality. In this study, we systematically confirmed the detrimental effects of oxidative stress on Nile tilapia muscle tissue, ranging from production traits to cells by establishing a chronic hydrogen peroxide (HP) exposure model. Additionally, we revealed the role of the Pten gene in this process through the integration of physiological and biochemical analyses, transcriptomic profiling, and cellular functional validation. This investigation provides a new theoretical basis for an in-depth understanding of the mechanisms underlying muscle oxidative damage in aquatic animals and for the development of intervention strategies.

As a reactive oxygen species, hydrogen peroxide can diffuse across cell membranes and trigger intracellular redox imbalance, leading to oxidative stress or oxidative damage [25,26]. To maintain redox homeostasis, fish activate a complex antioxidant defense system upon exposure to oxidative stress [27]. However, prolonged or severe oxidative stress can deplete antioxidants, compromise the antioxidant defense system, and ultimately lead to cell death or tissue damage [28]. Indeed, several previous studies have demonstrated that acute hydrogen peroxide stimulation reduces antioxidant parameters and impairs the antioxidant defense system in the liver or intestines of fish [19,29]. The present study indicated that chronic H_2_O_2_ treatment induced a sustained redox imbalance, characterized by a significant accumulation of MDA, a terminal product of lipid peroxidation, as well as a transition of the antioxidant enzyme system from an initial compensatory enhancement to a final state of depletion, revealing the progressive failure of the endogenous antioxidant defense system under chronic stress.

This persistent oxidative stress directly led to reductions in both body weight and muscle yield in Nile tilapia. This was closely associated with the downregulation of MyoG and the upregulation of Mstn. The myogenic regulatory factors (MRFs) family, including MyoD and MyoG, is critical for determining myoblast proliferation and differentiation [30]. Concurrently, the upregulation of Mstn, a potent negative regulator of muscle growth, can further exacerbate muscle atrophy by inhibiting myoblast proliferation and activating protein degradation pathways [31], indicating that oxidative stress disrupted normal proliferation and differentiation processes. In addition, the extracellular matrix (ECM) receptor-mediated signaling pathway is recognized as a critical regulatory node in maintaining skeletal muscle homeostasis. This pathway not only protects muscle fibers from excessive mechanical damage but also precisely directs the processes of repair and remodeling following injury [32]. However, under conditions of oxidative stress, excessive accumulation of ROS led to a homeostatic imbalance in the ECM–receptor signaling pathway, ultimately compromising the structural integrity and physiological function of muscle.

Importantly, oxidative stress significantly degraded the eating quality of the muscle, manifested as reductions in hardness, chewiness, and springiness. Hardness is a key dimension of meat texture; for instance, Wang et al. [33] demonstrated that increasing muscle hardness can improve the flesh quality of grass carp. Springiness is also an important indicator for evaluating the vitality of fish meat texture, and its reduction not only diminishes taste and freshness but also impairs processing performance [34]. Chewiness refers to the energy required to masticate solid food, and its decrease negatively affects the taste of the meat [35]. Therefore, the deterioration of tilapia muscle quality following H_2_O_2_ treatment directly impacts consumer sensory acceptance and product value. Histological and ultrastructural observations provided morphological evidence for this degradation: myofiber atrophy, loose arrangement, and disruption of sarcomere structure. These microscopic changes form the basis for the deterioration in meat quality traits. Coarse myofibrils in muscle fibers, characterized by thickness and tight cross-linking, typically result in higher hardness [36]. Greater fiber density contributes to a more delicate texture [37]. Longer fibers can increase the difficulty of fiber rupture during chewing, slightly enhancing chewiness [35]. Dense and regularly arranged muscle fiber bundles lead to good springiness and uniform hardness. Conversely, excessive inter-fiber spacing results in loose muscle structure and poor springiness [38]. Thus, the structural disorganization observed at the histological level provides a direct mechanistic explanation for the reduced hardness, springiness, and chewiness detected in texture analysis.

Further analysis indicated that chronic oxidative stress altered muscle cell fate. A significant upregulation of autophagy marker genes suggested the initiation of autophagic processes. Studies have shown that in the early stages of stress, moderate autophagy plays a protective role in clearing damaged organelles and maintaining intracellular homeostasis [39]. However, in this study, the sustained activation of autophagy might have accelerated protein loss and promoted muscle atrophy. Concurrently, the apoptosis was also significantly activated. ROS disrupt the mitochondrial membrane potential, promoting the release of cytochrome c and activating the caspase cascade, ultimately leading to programmed cell death [40]. Particularly noteworthy was the accompanying inflammatory response, characterized by significantly elevated pro-inflammatory cytokines and the transcription factor. Thus, oxidative stress evoked the muscle inflammatory response. Numerous studies have shown that oxidative stress and antioxidant inhibition are the underlying factors [41]. Similarly, correlation analysis further demonstrated that the collapse of the antioxidant defense system served as the critical trigger for initiating these cellular damage programs.

The phosphatase and tensin homolog (Pten) encodes a dual-specificity lipid and protein phosphatase and was initially identified as a tumor suppressor gene frequently mutated in various malignancies [42]. Based on transcriptomic and correlation analyses, Pten was identified as a key target under H_2_O_2_ exposure. As a core negative regulator of the PI3K/AKT signaling pathway, Pten inhibits AKT activity, leading to the dephosphorylation and activation of its downstream transcription factor, FOXO. Activated FOXO translocates to the nucleus and initiates the gene expression of various antioxidant enzymes (e.g., superoxide dismutase SOD, catalase), thereby enhancing the cellular antioxidant capacity and clearing excess reactive oxygen species (ROS) [43]. In this study, we found that the antioxidant NAC, while alleviating oxidative stress, significantly downregulated the expression of Ptenb, suggesting an association between Pten and the regulation of intracellular redox status. Research has shown that in pancreatic β cells, NAC inhibits the upregulation of Pten protein levels induced by cytokines by clearing peroxynitrite [44]. Further studies revealed that activating Pten reduced intracellular ROS levels and alleviated the cell proliferation inhibition. Conventionally, Pten activation is crucial for maintaining the quiescence of muscle stem cells, preventing excessive proliferation, and coordinating proper muscle fiber growth [45]. However, our results suggest that when cells are confronted with persistent oxidative stress, restoring or enhancing Pten activity to maintain antioxidant balance became a higher priority. The significantly inhibited expression of apoptosis- and inflammation-related genes further corroborated this conclusion. Although Pten was involved in regulating antioxidantion, the underlying mechanism warrants further investigation. In addition to mitigating cellular and physiological damage, activation of the antioxidant system held significant implications for the food industry. Previous studies have indicated that the pre-slaughter antioxidant status of muscle was a critical determinant of post mortem fish quality and shelf life. Impaired antioxidant capacity accelerated oxidative deterioration, leading to off odors, color changes, texture softening, and nutrient loss [46]. Conversely, enhancing the antioxidant capacity played a key role in retarding lipid and protein oxidation during cold storage. Therefore, the role of Pten in regulating antioxidant capacity might contribute to extending post mortem shelf life and reducing economic losses during processing and storage.

## 5. Conclusions

In summary, this study established an oxidative stress model in Nile tilapia via prolonged hydrogen peroxide exposure, elucidating the detrimental effects and regulatory mechanisms of oxidative stress on muscle health. Our findings demonstrate that persistent oxidative stress overwhelmed the antioxidant defense system, leading to growth retardation and muscle impairment. Furthermore, oxidative stress disrupted muscle cell fate and potentiated inflammatory responses in the muscle. Mechanistically, the activation of Pten was found to mitigate oxidative stress by enhancing the antioxidant potential to promote cell proliferation. This study highlights Pten as a potential therapeutic target for alleviating oxidative stress injury in aquaculture.

## Data Availability

The original contributions presented in this study are included in the article/Supplementary Materials. The data on RNA-seq are available at the Sequence Read Archive (SRA-NCBI) under the accession number PRJNA1444324. Further inquiries can be directed to the corresponding authors.

## Supplementary Materials

The following supporting information can be downloaded at https://www.mdpi.com/article/10.3390/antiox15040499/s1, Table S1: Primers and sequences referred in the experiment. References [47,48,49,50,51] are cited in the Supplementary Materials.
