# Supplementary material for: Pten-Mediated Antioxidant Response Alleviates Hydrogen Peroxide-Induced Oxidative Damage in Tilapia Muscle
Source: Antioxidants (Basel). 2026 Apr 17;15(4):499. doi: 10.3390/antiox15040499 (PMC13114245; doi:10.3390/antiox15040499)
Supplement: Supplementary file 1 [file antioxidants-15-00499-s001.zip › antioxidants-4236199-supplementary.pdf]

Table S1 Primers and sequences referred in the experiment.

Gene	Primer	Sequence (5' → 3')	Accession numbers	Gene	Primer	Sequence (5' → 3')	Accession numbers
On_Nrf2	F	TGGACCTGATTGACATACT	XM_031743127.2	*Zb_IL-1β*	F	CCACGTATGCGTCGCCCAGT	[48]
	R	TCTTTCTGACGGTGGTT			R	GGGCAACAGGCCAGGTACAG	
On_Sod	F	GACGTGACAACACAGGTTGC	XM_080289631.1	*Zb_Tnfα*	F	AGAAGGAGAGTTGCCTTTAC	[48]
	R	TACAGCCACCGTAACAGCAG			R	AACACCCTCCATACACCCGA	
On_Cat	F	CAACATGCCACCAGGAA	XM_019361816.2	*Zb_NFkB1*	F	GGCAGGTGGCGATAGTGTT	[48]
	R	CACATCGGACCATCACG			R	CATTCCTTCAGTTCTCTTGC	
On_MyoD	F	CGACGGCATGACGGATTTTA	XM_005449137	*Zb_Casp9*	F	AAATACATAGCAAGGCAACC	[49]
	R	GCTGCTGTTATCGGTGGAGA			R	CACAGGGAATCAAGAAAGG	
On_Myf5	F	CTGCTCTGATGGCATGGCTG	XM_005456634	*Zb_Bcl2*	F	TCACTCGTTCAGACCCTCAT	[49]
	R	CACGATACTGGACAGGCACT			R	ACGCTTTCCACGCACAT	
On_MyoG	F	TCGTCAAGCGAGCCCAGTT	NM_001279526.1	*Zb_Bax*	F	GGCTATTTCAACCAGGGTTC	[49]
	R	GTCAAGGCCCGCATGTTCC			R	TGCGAATCACCAATGCTGT	
On_Mstna	F	GACTTTGAGGAGTTCGGTTG	XM_003446535.5	*Zb_Casp3*	F	CCGCTGCCCATCACTA	[49]
	R	CCCGTGGGTTAGCCTTGTT			R	ATCCTTTCACGACCATCT	
On_Mstnb	F	TGAACCTGATTCCGCTGTCC	XM_003458832	*Zb_Ptena*	F	CCAGCCAGCGCAGGTATGTG	[50]
	R	GTCGATCTTCAAGGAGCGGA			R	GCGGCTGAGGAAACTCGAAG	
On_ATG5	F	TTATGTGCCACTGGGAGCAG	[47]	*Zb_Ptenb*	F	GCTACCTTCTGAGGAATAAG	[50]
	R	CTCACCCTGGGTAACAGCAG			R	CTTGATGTCCCCACACACAG	
On_ATG7	F	ACCCTGTTTCCGTACGGTTC	[47]	*Zb_Sod1*	F	GTTTCCACGTCCATGCTTT	NM_131294.1
	R	ACGTACCGTCAGTTTTCGCT			R	TCACATTACCCAGGTCTCC	
On_Beclin1	F	TTTCACATTTGGCACAGCGG	XM_005471281.3	*Zb_Cat*	F	AGCCACGCTTCCTTGAGT	NM_130912.2
	R	ATCTCTGAAAGTGCAGCCCC			R	CGGCTTCATTTAGCACCT	
On_LC3	F	CTGGTGGCAGACCATGTCAA	MH048880.1	*Zb_Foxo1*	F	ACACCTGGAGAAGCTACCGA	NM_001077257.2
	R	GACCGTTACCCAGCAGGAAA			R	AAGTCCAGCGATTCTCCGTC	
On_Bax	F	CCTCCAAAGAATGATAAATG	XM_019357746.2	*Zb_Nrf2*	F	AGATGAGAACGGAAAGGC	AB081314.1
	R	AGTAGAACAGGGCAACCA			R	AAGGCGAGGAACTAGGAA	
On_Bcl2	F	ATCGCAGACTGGATGACG	XM_003437902.5	*Zb_β-actin*	F	CCCTGAATCCCAAAGCCAA	[48]
	R	GGTATGCTCCGATGGTGA			R	CCATCACCAGAGTCCATCAC	
On_Casp3	F	AACCTGGATGATGGAGTC	XM_005456373.4	*On_IL-1β*	F	ACAAGGATGACGACAAGCCA	XM_019365841.2
	R	GGAATAGGCGTATAAGAAGT			R	GGACAGACATGAGAGTGCTG	
On_ Casp9	F	AGCATTGCCCATCTTTG	XM_025901776.1	*On_IL-6*	F	ACAGAGGAGGCGGAGATG	XM_019365841.2
	R	GGAATAGCGTCCGTCTG			R	GCAGTGCTTCGGGATAGAG	
On_NFkB1	F	GCAGGATTACGAGCCTTGGA	XM_003440691.4	*On_Tnfα*	F	CCAGAAGCACTAAAGGCGAAGA	[51]
	R	ATTGAGGAACGGGTGATTGT			R	CCTTGGCTTTGCTGCTGATC	
On_PI3k	F	GTAAAGCCACAGGGTCTCGG	XM_003457930.5	*On_Akt1*	F	GGAAACCACCGGCACAGATA	XM_003447818.5
	R	AACACGTCGTACAGTGGTCC			R	CGATGGTGGTTACAGAAGGA	
On_Ptena	F	GCAGCAAAGAAAGCGACAG	XM_005474212.4	*On_Ptenb*	F	TGTGCGGAGCGACATTAC	XM_003449407.5
	R	GGTGAGAAGTAGCGGTTAG			R	CGCAGAACGGCTTTATCAG	
On_β-actin	F	CCACACAGTGCCCATCTACG	XM_003443127.5				

	R	CCACGCTCTGTCAGGATCTT

Note: The mRNA sequences for each gene were obtained from the transcriptome sequencing database which was preserved in the lab. Primers were designed using Primer Premier 5.0.

47. Ibrahim, R.E., Elshopakey, G.E., Aly, Mohamed Y.M., Abdelwarith, A.A., Younis, E.M., Abd-Elhakim, Y.M., Khamis, T., Osman, A., Metwally, Mohamed M.M., Davis, S.J., Mohamed, A.A. Camel whey protein hydrolysate diet mitigates alkaline stress–induced biochemical disorders and restores the target of rapamycin, MAPK pathway, and autophagy-related gene expression in Nile tilapia. *Aquacult Int* 2024, 32, 9911-9932.
48. Wang, C., Chen, Y., Bian, W., Xie, S., Qi, G., Liu, L., Strauss, P.R., Zou, J., Pei, D. Deletion of mstna and mstnb impairs the immune system and affects growth performance in zebrafish. *Fish Shellfish Immunol* 2018, 72, 572-580.
49. Deng, J., Yu, L., Liu, C., Yu, K., Shi, X., Yeung, L., Lam, P., Wu, R., Zhou, B. Hexabromocyclododecane-induced developmental toxicity and apoptosis in zebrafish embryos. *Aquat Toxicol* 2009, 93, 29-36.
50. Croushore, J.A., Blasiole, B., Riddle, R.C., Thisse, C., Thisse, B., Canfield, V.A., Robertson, G.P., Cheng, K.C., Levenson, R. Ptena and ptenb genes play distinct roles in zebrafish embryogenesis. *Dev Dyn* 2005, 234, 911-21.
51. Elbahnaswy S., Elshopakey G.E. Differential gene expression and immune response of Nile tilapia (*Oreochromis niloticus*) challenged intraperitoneally with *Photobacterium damselae* and *Aeromonas hydrophila* demonstrating immunosuppression. Aquaculture, 2020, 526, 735364.
